# What is the best way to determine bond-valence parameters?

**DOI:** 10.1107/S2052252517011782

**Published:** 2017-09-01

**Authors:** I. David Brown

**Affiliations:** a Brockhouse Institute for Materials Research, McMaster University, Hamilton, ON, Canada L8S 4M1

**Keywords:** bond-valence method, coordination numbers, crystal radii, bond-valence parameters, bond softness

## Abstract

Two recent systematic determinations of bond-valence parameters addressed the problem of the correlation between *R*_0_ and *b* in different ways raising the question of which is to be preferred.

Pauling (1929 ▸) was the first to point out that ions distribute their valence charge among the bonds that they form. Later he showed that the amount of valence, *s*, used by each bond is correlated with its length, *R* (Pauling, 1947 ▸). In the past 70 years the empirical parameters describing this correlation, *R*_0_ and *b* in equation (1), have been determined for most of the bonds that one is likely to encounter,
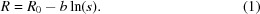
These empirical bond-valence parameters are fully transferable between all instances of the same bond type. *R*_0_ is the notional length of a bond of unit valence and *b* measures the softness of the interaction between the atoms. Values of (*R*_0_, *b*) are usually determined by minimizing the standard deviation, σ(Δ*V*), of the differences between the atomic valence and the sum of the bond valences around the same cation found in many well determined crystal structures. Recently Gagné & Hawthorne (2015 ▸) and Chen & Adams (2017 ▸) have independently published systematic studies of these parameters for many bond types. Gagné & Hawthorne’s (2015 ▸) study is restricted to oxides, but otherwise both studies are similar, carefully selecting a series of reliable crystal structures and refining (*R*_0_, *b*) for each bond type by minimizing σ(Δ*V*).

Two difficulties arise in this refinement. The first is the strong correlation between *R*_0_ and *b*, shown in Fig. 1 ▸ [Fig. 11 in Chen & Adams (2017 ▸)], that plots the standard deviation of σ(Δ*V*) as a function of *R*_0_ and *b* for Hg—Cl bonds. The regions in red represent acceptable values. Any value of *b* lying between 0.2 and 0.9 Å can give acceptable bond-valence sums providing the appropriate value of *R*_0_ (lying between 2.3 and 1.7 Å, respectively) is used. In their earlier tabulation of bond-valence parameters, Brown & Altermatt (1985 ▸) adopted a fixed value of *b* = 0.37 Å and this convention has been widely adopted in subsequent studies, but more recent work, notably by Adams (2001 ▸), has shown that larger values of *b* are needed when one of the atoms is soft in the Pearson (1973 ▸) sense.

The second difficulty is deciding how many bonds belong in the first coordination sphere, since the more bonds that are included, the larger the refined value of *b* and the smaller that of *R*_0_. For most cation environments the coordination number can be unambiguously assigned, but problems arise when the bonding is irregular, *e.g.* around cations with lone pairs which typically have a small number of primary bonds and several longer secondary bonds. In such cases, the choice of the cut-off distance strongly affects the resulting bond-valence parameters.

Compounding these difficulties are the uncertainties in the bond length measurements used to determine the bond-valence parameters, and the internal strains found in many compounds resulting from misfits in the sizes of atoms. Such strains are difficult to detect when selecting structures for study, leading to uncertainties of several hundredths of an ångström in the bond lengths.

Gagné & Hawthorne (2015 ▸) optimized both *R*_0_ and *b* simultaneously and stated that they included both primary and secondary bonds for cations with stereoactive lone pairs. Only later did they give a more complete discussion of the coordination number (Gagné & Hawthorne, 2016 ▸). Their goal was to find the values of (*R*_0_, *b*) that gave the lowest value of σ(Δ*V*), but the true location of this minimum is not known since it depends on the experimental bond lengths used in the calculation. Consequently, Gagné & Hawthorne’s (2015 ▸) parameters show a scatter that makes it more difficult to see the systematic trends.

When working with amorphous materials where a coordination number cannot be defined, Adams (2001 ▸) chose coordination spheres with a cut-off beyond which the refined values of (*R*_0_, *b*) do not change, typically around 6 Å. He also noted that *b* increased with an increase in the difference between the Pearson softness of the two bonded atoms. Using values of *b* calculated from the Pearson softness he produced the *softBV* set of (*R*_0_, *b*) parameters suitable for work with amorphous structures (Adams, 2017 ▸). As expected, these values of (*R*_0_, *b*) are significantly different from those calculated using only the first coordination sphere. Believing that the *softBV* parameter *b* was more physically meaningful than either Brown & Altermatt’s (1985 ▸) 0.37 Å or the refined values of Gagné & Hawthorne (2015 ▸), Chen & Adams (2017 ▸) report a new set of parameters with values of *b* calculated using the Pearson softness and values of *R*_0_ calculated using distances from just the first coordination sphere. For greater consistency they define the first coordination sphere as including all the bonds with valences, *s*, that satisfy the inequality
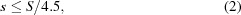
where *S* is the mean bond valence in each first coordination sphere, a definition that may exclude some secondary bonds. The resulting set of (*R*_0_, *b*) parameters for 706 different bond types are included in Chen & Adams’ (2017 ▸) supporting information.

Competing with Chen & Adams’ (2017 ▸) values are both the Gagné & Hawthorne (2015 ▸) parameters refined to give the best statistical fit without introducing chemical considerations, and the more traditional values mostly refined with *b* fixed at 0.37 Å often with unspecified first coordination spheres (Brown, 2017 ▸). Comparisons between the values of σ(Δ*V*) for the different sets show that they all provide acceptable bond-valence sums around the cations as long as the appropriate definition of cation coordination number is used. It will be interesting to see which set finds the greatest favour and whether the publication of these parameters will stimulate further efforts to understand the true nature of the bond length/bond valence correlation.

## Figures and Tables

**Figure 1:**
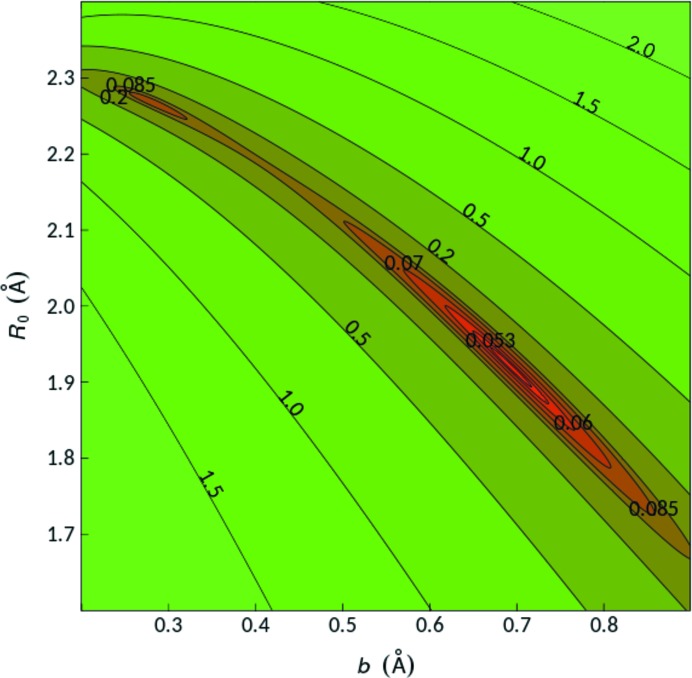
Colour-coded projection of the Δ*V* landscape as a function of *R*_0_ and *b* for our Hg^2+^—Cl^−^ reference data set, which contains *n* = 13 cation environments. From Chen & Adams (2017 ▸).
